# Relationship between astrocyte reactivity, using novel ^11^C-BU99008 PET, and glucose metabolism, grey matter volume and amyloid load in cognitively impaired individuals

**DOI:** 10.1038/s41380-021-01429-y

**Published:** 2022-02-07

**Authors:** Nicholas R. Livingston, Valeria Calsolaro, Rainer Hinz, Joseph Nowell, Sanara Raza, Steve Gentleman, Robin J. Tyacke, Jim Myers, Ashwin V. Venkataraman, Robert Perneczky, Roger N. Gunn, Eugenii A. Rabiner, Christine A. Parker, Philip S. Murphy, Paul B. Wren, David J. Nutt, Paul M. Matthews, Paul Edison

**Affiliations:** 1 Department of Brain Sciences, Imperial College London, London, UK; 2 Wolfson Molecular Imaging Centre, University of Manchester, Manchester, UK; 3 Department of Psychiatry and Psychotherapy, University Hospital, LMU Munich, Munich, Germany; 4 German Centre for Neurodegenerative Disorders (DZNE), Munich, Germany; 5 Munich Cluster for Systems Neurology (SyNergy), Munich, Germany; 6 Ageing Epidemiology Research Unit (AGE), School of Public Health, Imperial College London, London, UK; 7 Invicro, London, UK; 8 King’s College London, London, UK; 9 GlaxoSmithKline, Stevenage, UK; 10 UK Dementia Research Institute at Imperial College London, London, UK

**Keywords:** Biomarkers, Neuroscience

## Abstract

*Post mortem* neuropathology suggests that astrocyte reactivity may play a significant role in neurodegeneration in Alzheimer’s disease. We explored this in vivo using multimodal PET and MRI imaging. Twenty subjects (11 older, cognitively impaired patients and 9 age-matched healthy controls) underwent brain scanning using the novel reactive astrocyte PET tracer ^11^C-BU99008, ^18^F-FDG and ^18^F-florbetaben PET, and T1-weighted MRI. Differences between cognitively impaired patients and healthy controls in regional and voxel-wise levels of astrocyte reactivity, glucose metabolism, grey matter volume and amyloid load were explored, and their relationship to each other was assessed using Biological Parametric Mapping (BPM). Amyloid beta (Aβ)-positive patients showed greater ^11^C-BU99008 uptake compared to controls, except in the temporal lobe, whilst further increased ^11^C-BU99008 uptake was observed in Mild Cognitive Impairment subjects compared to those with Alzheimer’s disease in the frontal, temporal and cingulate cortices. BPM correlations revealed that regions which showed reduced ^11^C-BU99008 uptake in Aβ-positive patients compared to controls, such as the temporal lobe, also showed reduced ^18^F-FDG uptake and grey matter volume, although the correlations with ^18^F-FDG uptake were not replicated in the ROI analysis. BPM analysis also revealed a regionally-dynamic relationship between astrocyte reactivity and amyloid uptake: increased amyloid load in cortical association areas of the temporal lobe and cingulate cortices was associated with *reduced* ^11^C-BU99008 uptake, whilst increased amyloid uptake in primary motor and sensory areas (in which amyloid deposition occurs later) was associated with *increased* ^11^C-BU99008 uptake. These novel observations add to the hypothesis that while astrocyte reactivity may be triggered by early Aβ-deposition, sustained pro-inflammatory astrocyte reactivity with greater amyloid deposition may lead to astrocyte dystrophy and amyloid-associated neuropathology such as grey matter atrophy and glucose hypometabolism, although the evidence for glucose hypometabolism here is less strong.

## Introduction

Astrocytes are integral to normal brain function, playing important roles in neurogenesis, synaptogenesis, control of blood-brain barrier permeability and maintaining extracellular homeostasis [1]. In Alzheimer’s disease (AD), astrocytes can assume a reactive phenotype in response to disease by undergoing morphological, molecular and functional remodeling [2]. While astrocyte reactivity is associated with amyloid beta (Aβ) plaques [3], the precise role of this astrocyte reactivity in neurodegeneration is still unclear. It is suggested that astrocytes could have a beneficial and detrimental role, and this could also depend on the pathological insult and the susceptibility of the host [4]. It is proposed that with higher levels of Aβ, astrocyte reactivity can produce neurotoxic reactive oxygen species and inflammatory cytokines [5]. Astrocytes in AD also can lose normal neuroprotective capabilities as they become dystrophic with the progression of AD pathology [4, 6].

Glucose hypometabolism, measured using ^18^F-fluorodeoxyglucose (^18^F-FDG) PET, and brain atrophy, measured using MRI, are two of the earliest neuroimaging markers of neurodegeneration developed for AD [7]. However, both measures are sensitive to changes in astrocyte reactivity [8, 9], and the contribution of FDG signal from astrocyte reactivity is yet to be fully elucidated. By contributing to synaptic loss and neurodegeneration, pro-inflammatory and dystrophic astrocytes may be associated with accelerated grey matter (GM) atrophy [10]. Astrocytes are also necessary for metabolic support of neuronal activity [11], so AD-related changes in astrocytes might contribute directly to the brain glucose hypometabolism characteristic of AD [7].

The novel PET tracer ^11^C-BU99008 has high specificity and selectivity for binding sites of type-2 imidazoline receptors (I_2_-BS), which are expressed primarily within astrocytes and are upregulated with reactivity [12]. This tracer thus allows the study of astrocyte reactivity in vivo [13–18]. Pathologically increased ^11^C-BU99008 PET signal recently has been demonstrated in neurodegenerative disorders including AD [19] and Parkinson’s disease [20]. Currently, the only available PET tracer which can measure astrocyte reactivity in vivo is ^11^C-deuterium-_L_-deprenyl (^11^C-DED) [21, 22]. However, this tracer binds to monoamine oxidase-B (MAO-B), which is reduced in the presence of late stage Aβ-deposition. Therefore, it remains unclear if the lower ^11^C-DED binding observed in late-stage compared to early-stage AD subjects reflects a reduction in astrocyte reactivity or simply lower levels of MAO-B [23]. The higher specific binding of ^11^C-BU99008 than ^11^C-DED to detect astrocyte reactivity has recently been demonstrated in *post mortem* brain tissue from AD patients [24]—likely due to the fact they will be detecting different astrocyte subtypes—and thus ^11^C-BU99008 warrants further study in this clinical population. The aim of this study was to evaluate the relationship between astrocyte reactivity, using ^11^C-BU99008 PET, and glucose metabolism, GM atrophy and Aβ-deposition in cognitively impaired patients with a clinical diagnosis of AD-related dementia or Mild Cognitive Impairment (MCI).

## Materials and methods

We recruited 20 subjects for this pilot study. Ethical approval was obtained from the local and regional Research Ethics Committee, whilst approval to administer radiotracers was obtained from the Administration of Radioactive Substances Advisory Committee UK. The human biological samples sourced from participants were obtained ethically and their research use was in accordance with the terms of the informed consent.

### Subjects

Subjects were recruited from memory clinics, research registries and advertisements. We included 11 cognitively impaired patients with a clinical diagnosis of AD-related dementia or MCI (6 clinically diagnosed AD, 5 MCI; Mini-Mental Status Examination (MMSE) score [mean ± SD] = 22.6 ± 4.1) and 9 age-matched healthy controls (MMSE score [mean ± SD] = 29.1 ± 1.27) without a history of brain disease (Table 1).  The inclusion criteria for cognitively impaired patients included the ability to give informed consent, an MMSE score ≥17 and at least 8 years of education. Exclusion criteria for all participants included contradictions to MRI and any evidence of significant small vessel or vascular disease on MRI. AD patients were defined according to a clinical diagnosis based on previous MRI and/or FDG PET, and Aβ imaging from this study was not used as an inclusion/exclusion criteria, nor were AD patients excluded who were Aβ-negative according to our analysis. All subjects underwent medical and detailed cognitive assessments using the Repeatable Battery for the Assessment of Neuropsychological Status (RBANS), as well as ^11^C-BU99008, ^18^F-FDG and ^18^F-florbetaben PET and T1-weighted structural MRI. Aβ-positivity was defined by using a whole brain uptake cut-off of 1.43 [25].

**Table 1:** Demographic information and cognitive scores.

	*N*	Sex (M:F)	Age (years) Mean (±SD)	Global Aβ PET SUVr Mean (±SD)	MMSE Mean (±SD)	Immediate Memory Mean (±SD)	Visuospatial Constructional Mean (±SD)	Language Mean (±SD)	Attention Mean (±SD)	Delayed Memory Mean (±SD)
*Healthy controls*	9	5:4	69.8 (8.5)	1.22 (0.07)	29.1 (1.3)	115.4 (11.0)	100.5 (16.0)	100.8 (6.3)	110.0 (12.7)	105.0 (7.8)
*Cognitively impaired*	11 (7 Aβ +, 4 Aβ−)	8:3	74.0 (4.5)	1.59 (0.32)	22.6 (4.1)	65.5 (20.0)	86.0 (20.6)	81.5 (15.4)	87.5 (19.0)	61.5 (17.3)
*P values*	N/A	N/A	0.919	0.001^a^	0.001^a^	<0.001^a^	0.172	0.003^a^	0.012	<0.001^a^

Table showing demographic information and scores from each sub-category of the RBANS for healthy controls and cognitively impaired patients.

*Aβ+*: amyloid positive, *Aβ−*: amyloid negative, *F*: female, *M*: male, *MMSE*: mini-mental state examination, *SD*: standard deviation, *SUVr*: standard uptake value ratio.

^a^=*p*  <  0.01 between cognitively impaired and healthy control groups.

### Image acquisition

All image acquisition was performed at the Invicro Centre for Imaging Sciences in London, UK. MRI images were acquired using either a 3 Tesla Magnetom Trio or Verio (Siemens Healthcare Sector, Erlangen, Germany) with a 32-receiver channel head matrix coil. All PET imaging was performed on a Siemens Truepoint PET/CT scanner. All three PET scans were completed on separate days, with no longer than 30 days between the first and last scan completed. The order of the PET scans was not fixed and varied between participants depending on the availability of the PET tracers.

### MRI

#### Structural MRI

All subjects underwent a sagittal T1-weighted MPRAGE, acquired with TR = 2400 ms, TE = 3.06 ms, flip angle = 9°, TI = 900 ms, matrix = [256 × 246], a 1 mm isotropic voxel size, anteroposterior phase encoding direction, IPAT factor 2 and a symmetric echo.

#### PET

##### ^11^C-BU99008 PET

All subjects underwent ^11^C-BU99008 PET scanning to assess astrocyte reactivity in the brain. ^11^C-BU99008 was synthesised on site. An initial CT scan was acquired for attenuation correction of the PET images, before a mean activity of 330 (±30) MBq ^11^C-BU99008 in 20 ml normal saline was injected into the antecubital vein. Dynamic emission ^11^C-BU99008 PET images were acquired over 120 min and rebinned into 29 timeframes: 8 × 15 s, 3 × 60 s, 5 × 120 s, 5 × 300 s, and 8 × 600 s. All subjects had arterial blood sampled continuously for the first 15 min, with 12 additional samples taken at 5, 10, 15, 20, 25, 30, 40, 50, 60, 70, 80, and 100 min after injection. A gamma counter was used to measure radioactivity in the whole blood and plasma for each sample. Reverse-phase high-performance liquid chromatography was used to evaluate metabolism of ^11^C-BU99008 by calculating the relative proportions of parent tracer and metabolites in the blood. Parametric images (Impulse Response Function at 120 min (IRF-120)) of ^11^C-BU99008 were generated using spectral analysis. This was performed using Modelling, Input Functions and Compartmental Kinetics Parametric Map (MICK-PM) software (available on request from Wolfson Molecular Imaging Centre, University of Manchester, Manchester, UK).

##### ^18^F-FDG and ^18^F-florbetaben PET

All subjects also underwent ^18^F-FDG and ^18^F-florbetaben PET scanning to assess glucose metabolism and Aβ-deposition in the brain, respectively. Subjects received a target dose of 185 MBq ^18^F-FDG and 236 MBq ^18^F-florbetaben as single intravenous boluses in the respective scanning sessions. For ^18^F-FDG scans, PET acquisition commenced 30 min after tracer injection, and the scans were acquired for 30 min. Using MICKPM, activity over the last 30 min was averaged, resulting in a 3D 30–60 min ^18^F-FDG add-image. For ^18^F-florbetaben scans, PET acquisition commenced 90 min after tracer administration and the subjects were scanned for 30 min. Activity over the 30 min acquisition period was averaged, resulting in a 3D 90–120 min ^18^F-florbetaben add-image.

### Image processing

MRI and PET images were pre-processed using SPM12 (Wellcome centre for human neuroimaging, UCL, London, UK) in MATLAB (v2014a). 3D PET data was co-registered to the structural MRI of each subject. The structural MRI was segmented into GM, white matter (WM) and cerebrospinal fluid, and the GM and WM maps were used to generate a study-specific template using Diffeomorphic Anatomical Registration Through Exponential Lie Algebra (DARTEL) [26]. The DARTEL flow fields were then used to normalise each of the co-registered PET images and GM maps to MNI space and an 8 mm FWHM Gaussian kernel was used to smooth the data. Tracer uptake for ^11^C-BU99008 PET was calculated through spectral analysis (IRF-120 min). Tracer uptake for ^18^F-FDG and ^18^F-florbetaben PET was evaluated using the standardised uptake value ratio (SUVr) and the Hammers atlas [27], referenced to the pons GM/WM, and the cerebellar GM, respectively. This was done by dividing the cerebral cortical ^18^F-FDG and ^18^F-florbetaben mean images by the uptake value of the relevant reference region, which had been calculated in Analyse 11.0 (developed by the Biomedical Imaging Resource at the Mayo Clinic). This resulted in smoothed, normalised, ^18^F-FDG, ^18^F-florbetaben, ^11^C-BU99008 and GM Voxel-Based Morphometry (VBM) images that were used to assess glucose metabolism, Aβ deposition, astrocyte reactivity and GM atrophy patterns, respectively. This was done through Regions of Interest (ROI) analysis, as well as voxel-wise Statistical Parametric Mapping (SPM) and Biological Parametric Mapping (BPM) analysis. Partial Volume Correction (PVC) was not performed as PVC can increase noise within the data [28], leading to further signal leakage between regions and tissue classes which cannot be corrected for [29]. In addition, when comparing in vivo Aβ PET within subject with *post mortem* slices in AD subjects, PVC has been shown to have either no effect [30] or reduce [31] the accuracy of quantification of Aβ. Furthermore, as we are looking at increases for both ^11^C-BU99008 and ^18^F-Florbetaben, the PVC would have augmented the effect.

### Statistical analysis

#### ROI analysis

Subject-specific object maps were created from the Hammers atlas [27, 32] and were used to sample the ROI radioactivity concentration for the three normalised (not smoothed) PET images, as well as the ROI volume of the VBM images. The ROIs included the frontal lobe, temporal lobe, medial temporal lobe, parietal lobe, occipital lobe, posterior cingulate and the whole brain (made up of the four lobes and the cingulate). Tracer uptake and GM volume for each ROI in cognitively impaired patients was compared against that of the healthy controls using a two-sample Student’s *t* test (two-tailed), with statistical difference set to *p* < 0.05. Due to the exploratory nature of this study, multiple comparison corrections were not performed. Correlation between each of the four imaging measures in each of the four lobes and whole brain for Aβ-positive patients was calculated using Pearson’s correlation coefficient in SPSS (v26, released 2019).

#### SPM analysis

Voxel-level SPM analysis was performed in order to better characterise the spatial distribution of tracer uptake difference between the cognitively impaired patients and the healthy controls. The three smoothed, normalised  PET  and VBM images of all subjects were entered into four separate two-sample Student’s *t* tests in SPM12 (two-tailed). Significant clusters were identified using cluster-level family wise error (FWE) corrected *p* values. Single subject analysis was also performed on each of the four modalities by comparing each patients’ images against a mean healthy control image in further separate two-sample Student’s *t* tests in SPM12 (two-tailed).

#### BPM correlation analysis

In order to assess the neuroanatomical relationship between ^11^C-BU99008 binding and glucose metabolism, Aβ deposition and GM atrophy, *Z*-score maps for each of the four imaging modalities were created. These represent tracer uptake and GM atrophy patterns relative to the healthy control’s mean and standard deviation for each subject on a voxel-level basis, calculated with the following formulae:

$$	{{{\rm{Zmap}}}}\,{{{\rm{of}}}}\,^{11}\,{{{\rm{C}}}} - {{{\rm{BU}}}}99008 = \frac{{{\rm{Patient}}}\,^{11}\,{{{\rm{C}}}} - {BU}99008 - {mean}\,{of}\,{controls}\,^{11}\,{C} - {BU}99008}{{{\rm{SD}}}\,{of}\,{controls}\,^{11}\,{C} - {BU}99008}$$

$${{{{{\rm{Zmap}}}}\,{{{\rm{of}}}}\,^{18}\,{{{{{\rm{F}}}} - {{{\rm{FDG}}}}}} =\frac{{{{{\rm{Patient}}}}\,^{18}\,{{{{{\rm{F}}}} - {{{\rm{FDG}}}}}} \!-\! {{{\rm{mean}}}}\,{{{\rm{of}}}}\,{{{\rm{controls}}}}\,^{18}\,{{{{{\rm{F}}}} -{{{\rm{FDG}}}}}}}}{{{{{\rm{SD}}}}\,{{{\rm{of}}}}\,{{{\rm{controls}}}}\,^{18}\,{{{{{\rm{F}}}} - {{{\rm{FDG}}}}}}}}}}$$

$$	{{\rm{Zmap}}}\,{of}\,^{18}\,{F} - {florbetaben} = \frac{{{\rm{Patient}}}\,^{18}\,{F} - {florbetaben} - {mean}\,{of}\,{controls}\,^{18}\,{F} - {florbetaben}}{{{\rm{SD}}}\,{of}\,{controls}\,^{18}\,{F} - {florbetaben}}$$

$${{{{{\rm{Zmap}}}}\,{{of}}\,{{{\rm{VBM}}}} = \frac{{{{{\rm{Patient}}}}\,{{{\rm{VBM}}}} - {{{\rm{mean}}}}\,{{{\rm{of}}}}\,{{{\rm{controls}}}}\,{{{\rm{VBM}}}}}}{{{{{\rm{SD}}}}\,{{{\rm{of}}}}\,{{{\rm{controls}}}}\,{{{\rm{VBM}}}}}}}}$$

Voxel-level correlations between ^11^C-BU99008 and the remaining three modalities were estimated for Aβ-positive patients using BPM [33], an SPM toolbox that runs through MATLAB and SPM5. Due to the exploratory nature of this study, multiple comparison corrections were not performed.

## Results

### ROI and single subject SPM analysis

**Fig. 1:**
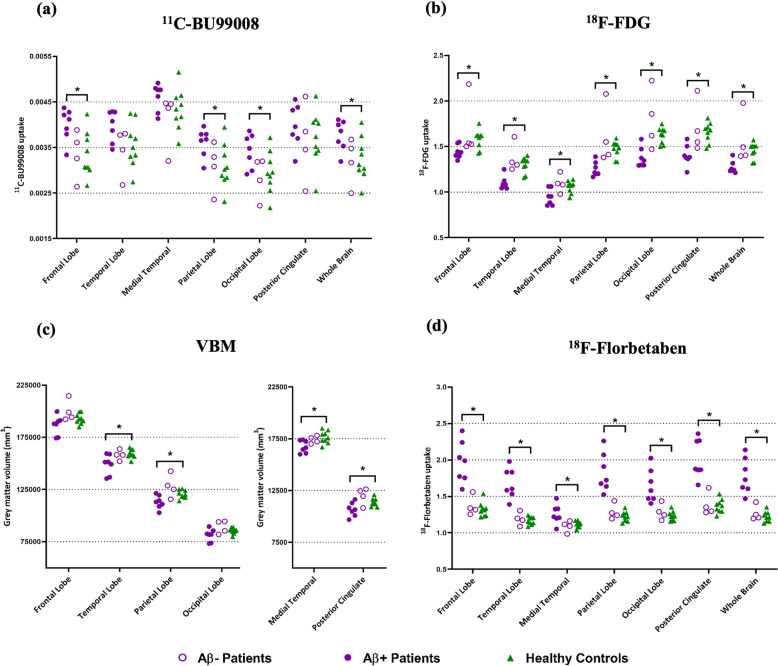
Region of Interest (ROI) analysis in all patients and healthy controls. Dot plots demonstrating regional changes in Aβ-positive patients (purple filled circle, *N* = 7), Aβ-negative patients (purple open circle, *N* = 4) and healthy controls (green triangle, *N* = 9): (**a**) ^11^C-BU99008 (**b**) ^18^F-FDG (**c**) VBM and (**d**) ^18^F-Florbetaben. Difference in tracer uptake and GM volume between Aβ-positive patients and healthy controls was analysed using two-sample Student’s *t* tests (two-tailed), with statistical difference set to *p* < 0.05. Multiple comparison corrections were not performed on the ROI results due to the exploratory nature of this pilot study. “Whole Brain” refers to the composite cortex, combining all the four lobes and the cingulate. * denotes *p* < 0.05.

All the healthy controls were Aβ-negative, 7 of the patients were Aβ-positive (4 AD, 3 MCI) and the other 4 patients were Aβ-negative (2 AD, 2 MCI). One of the patients classified as Aβ-positive in the previous publication [19] (whole-brain uptake of 1.43779) was classified as Aβ-negative in this publication following the DARTEL analysis which reduced the whole-brain uptake (1.42195) to beneath the Aβ-positivity threshold used (1.43 3sf). Global tracer uptake (mean uptake ± SD) of ^18^F-florbetaben was 1.55 ± 0.29 for patients and 1.21 ± 0.06 for controls. Shapiro–Wilk’s tests confirmed data of global tracer uptake were normally distributed for patients (W(11) = 0.948, *p* = 0.616) and controls (W(9) = 0.956, *p* = 0.751). Brain ^18^F-florbetaben uptake was increased in patients compared to healthy controls (*p* = 0.003; Fig. 1d), with regional increases significant in the frontal (*p* = 0.004), temporal (*p* = 0.004), medial temporal (*p* = 0.045) and parietal (*p* = 0.003) lobes, as well as the posterior cingulate (*p* = 0.005) and hippocampus (*p* = 0.042).

Global tracer uptake (mean uptake ± SD) of ^11^C-BU99008 was 82.7 ± 11.5 for patients and 77.7 ± 7.7 for healthy controls. Shapiro–Wilk’s tests confirmed data of global tracer uptake were normally distributed for patients (W(11) = 0.942, *p* = 0.546) and controls (W(9) = 0.869, *p* = 0.095). Aβ-positive patients showed increased ^11^C-BU99008 brain uptake compared to healthy controls (*p* = 0.021; Fig. 1a). Regional analyses showed increases particularly in the frontal (*p* = 0.007), parietal (*p* = 0.018), and occipital (*p* = 0.039) lobes. An exploratory two-sample Student’s *t* test was run comparing MCI and AD subjects, demonstrating increased ^11^C-BU99008 uptake in MCI patients in the anterior (*p* = 0.023) and posterior (*p* = 0.007) cingulate.

Global tracer uptake (mean uptake ± SD) of ^18^F-FDG was 1.38 ± 0.22 for patients and 77.7 ± 7.7 for controls. Shapiro–Wilk’s tests confirmed data of global tracer uptake were normally distributed for patients (W(11) = 0.874, *p* = 0.114) and controls (W(9) = 0.087, *p* = 0.137). Aβ-positive patients showed decreased ^18^F-FDG brain uptake compared to healthy controls (*p* = 0.021; Fig. 1b). Regional analysis showed decreases in the frontal (*p* = 0.009), temporal (*p* < 0.001), medial temporal (*p* = 0.018), parietal (*p* < 0.001) and occipital (*p* < 0.001) lobes, as well as the posterior cingulate (*p* < 0.001) and hippocampus (*p* < 0.018).

Whole brain volume (mean volume ± SD) was 568272 ± 37709 for patients and 580365 ± 14566 for controls. Shapiro–Wilk’s tests confirmed data of whole brain volume were normally distributed for patients (W(11) = 0.963, *p* = 0.856) and controls (W(9) = 0.929, *p* = 0.513). Aβ-positive patients had lower whole brain GM volume than the healthy controls (*p* = 0.046; Fig. 1c). Regionally, decreases were significant in the temporal (*p* = 0.029), medial temporal (*p* = 0.009) and parietal (*p* = 0.020) lobes, as well as the posterior cingulate (*p* = 0.036) and hippocampus (*p* = 0.032).

**Fig. 2:**
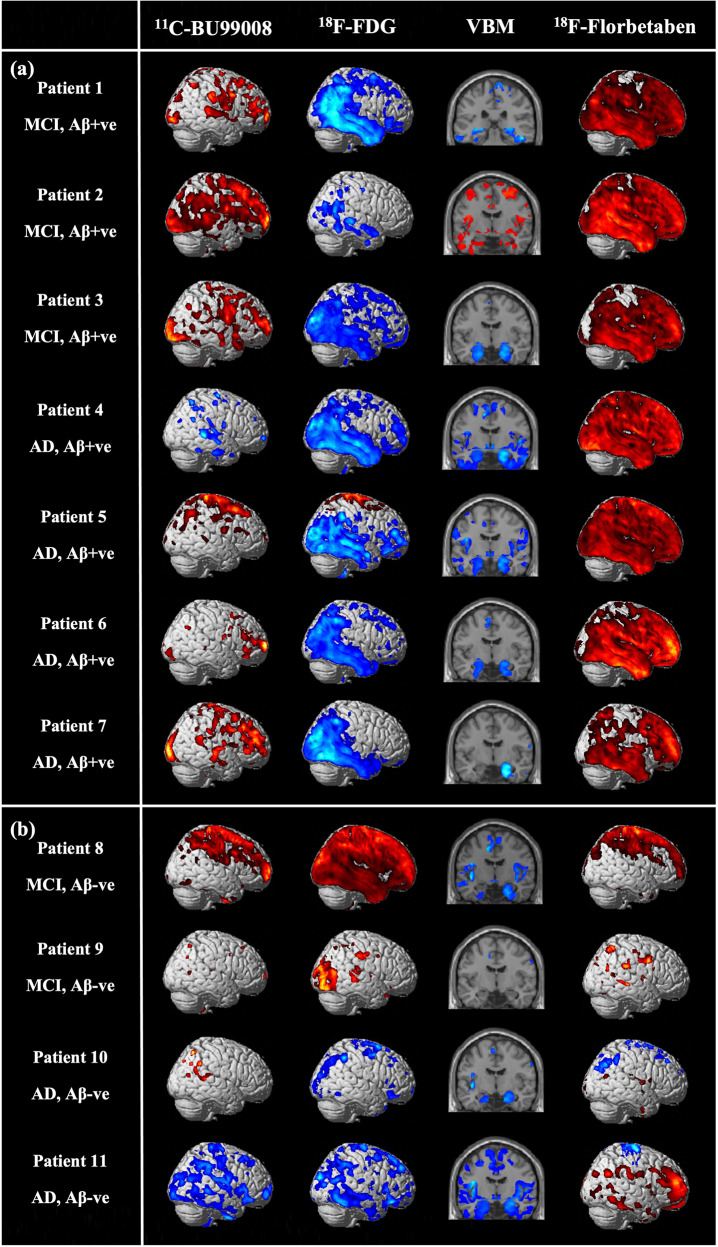
Single subject Statistical Parametric Mapping (SPM) analysis of ^11^C-BU99008, ^18^F -FDG, VBM and ^18^F-Florbetaben. Rendered single subject SPM results from two-sample Student’s *t* tests (two-tailed) for differences in tracer uptake and GM volume between each patient and healthy controls (*N* = 9): (**a**) Single subject SPM analysis of Aβ-positive patients for ^11^C-BU99008, ^18^F -FDG, VBM and ^18^F-Florbetaben, rendered at cluster threshold of *p* < 0.05 with an extent threshold of 50 voxels. **b** Single subject SPM analysis of Aβ-negative patients for ^11^C-BU99008, ^18^F -FDG, VBM and ^18^F-Florbetaben, rendered at cluster threshold of *p* < 0.05 with an extent threshold of 50 voxels.

SPM single subject analysis showed inter-subject and inter-regional heterogeneity in ^11^C-BU99008 uptake, and consistently reduced ^18^F-FDG uptake and GM atrophy in the temporal lobe and hippocampus of Aβ-positive patients (Fig. 2).

Please note, the ROI and single subject SPM results were not corrected for multiple comparisons due to the exploratory nature of the study.

### Group-level SPM analysis

**Fig. 3:**
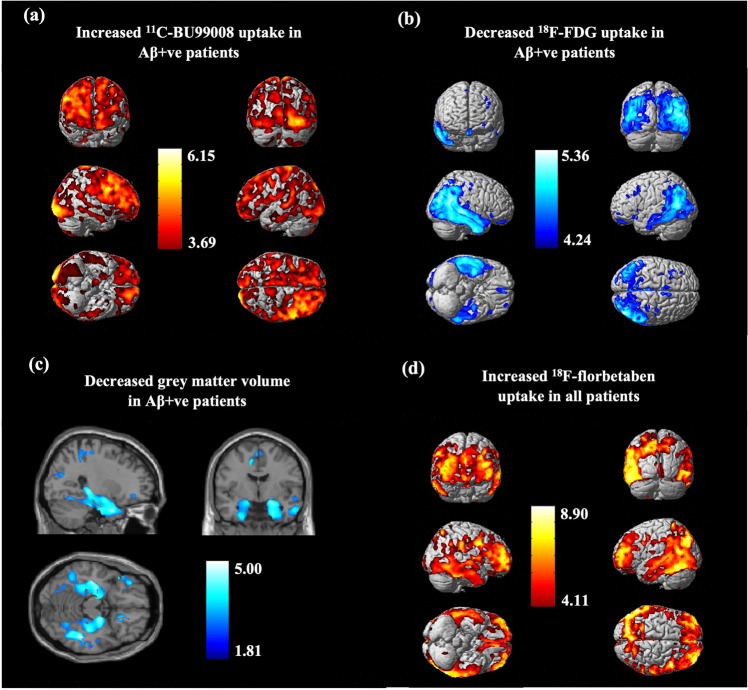
Statistical Parametric Mapping (SPM) group analysis in patients compared to healthy controls. Rendered SPM results from two-sample Student’s *t* tests (two-tailed) for differences in tracer uptake and GM volume between patients (*N* = 11) and healthy controls (*N* = 9): (**a**) Increased ^11^C-BU99008 uptake in Aβ-positive patients (*N* = 7), rendered at cluster threshold of *p* < 0.05 and an extent threshold of 50 voxels. **b** Decreased ^18^F-FDG uptake in Aβ-positive patients (*N* = 7), rendered at cluster threshold of *p* < 0.001 and an extent threshold of 50 voxels. **c** Decreased GM volume in Aβ-positive patients (*N* = 7), rendered at cluster threshold of *p* < 0.05 and an extent threshold of 50 voxels. **d** Increased ^18^F-Florbetaben uptake in all patients (*N* = 11), rendered at cluster threshold of *p* < 0.001 and an extent threshold of 50 voxels. Coordinates for significant clusters following FWE-correction can be found in Supplementary Table 1. Colourbar units are contrast estimates representative of *Z*-scores.

Two-sample Student’s *t* test in SPM contrasting Aβ-positive patients and healthy controls showed distributions of differences in tracer uptake and GM volumes that were consistent with the ROI-analyses (Fig. 3 and Supplementary Table 1). Following cluster-level FWE-correction, Aβ-positive patients had increased ^11^C-BU99008 uptake particularly in the frontal and occipital lobes (Fig. 3a), reduced ^18^F-FDG uptake in the temporal, parietal and occipital lobes (Fig. 3b), reduced GM volume in temporal regions, particularly the hippocampi (Fig. 3c), and increased ^18^F-florbetaben uptake in frontotemporal regions (Fig. 3d). An exploratory two-sample Student’s *t* test comparing MCI and AD subjects showed increased ^11^C-BU99008 uptake in MCI patients, particularly in the frontal and temporal regions (Supplementary Fig. 1 and Supplementary Table 1).

### Regional and voxel-wise correlations

#### Associations of ^11^C-BU99008 and ^18^F-FDG

**Fig. 4:**
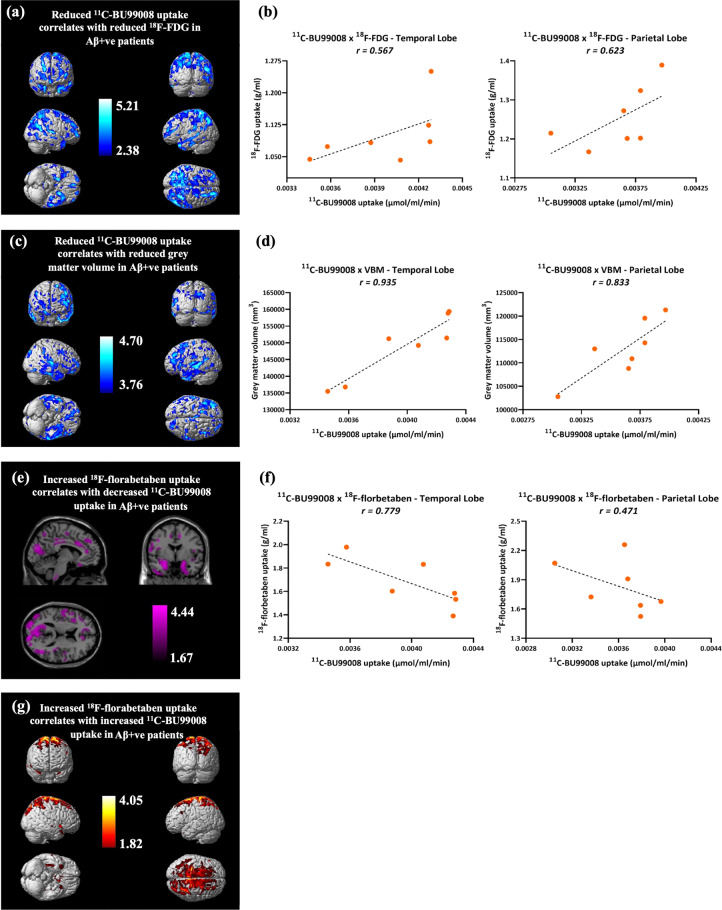
Biological Parametric Mapping (BPM) and Region of Interest (ROI) correlations of ^11^C-BU99008 with ^18^F-FDG, VBM and ^18^F-Florbetaben in Aβ-positive patients (*N* = 7). **a** *BPM*: Reduced ^11^C-BU99008 uptake correlated with reduced ^18^F-FDG uptake, rendered at cluster threshold of *p* < 0.01. **b** *ROI:* ^11^C-BU99008 uptake positively correlated with ^18^F-FDG uptake in the temporal (*r* = 0.567) and parietal (*r* = 0.623) lobes, although these did not reach significance (*p* = 0.184 and *p* = 0.135, respectively). **c** *BPM:* Reduced ^11^C-BU99008 uptake correlated with reduced GM volume, shown through sections at cluster threshold of *p* < 0.01. **d** *ROI*: ^11^C-BU99008 uptake positively correlated with GM volume in the temporal (*r* = 0.935, *p* = 0.002) and parietal (*r* = 0.833, *p* = 0.02) lobes. **e** *BPM:* Increased ^18^F-florbetaben uptake correlated with reduced ^11^C-BU99008 uptake, rendered at cluster threshold of *p* < 0.05. **f** *ROI:* ^11^C-BU99008 uptake positively correlated with ^18^F-florbetaben uptake in the temporal (*r* = 0.779) and parietal (*r* = 0.471) lobes, although only the temporal lobe reached significance (*p* = 0.039 and *p* = 0.287, respectively). **g** *BPM:* Increased ^18^F-florbetaben uptake correlated with increased ^11^C-BU99008 uptake, rendered at cluster threshold of *p* < 0.05. Colourbar units in (**a**), (**c**), (**e**), and (**g**) are contrast estimates representative of *Z*-scores. All BPM correlations are displayed with an extent threshold of 50 voxels. Multiple comparison corrections were not performed on the BPM results due to the exploratory nature of this pilot study.

BPM analysis in Aβ-positive patients showed that on a voxel-wise basis reduced ^11^C-BU99008 uptake was associated with reduced ^18^F-FDG uptake, particularly in the parietal, frontal and temporal lobes (Fig. 4a). However, these results were not replicated within the ROI correlation analyses (frontal (*r* = 0.202, *p* = 0.664), occipital (*r* = 0.264, *p* = 0.567), temporal (*r* = 0.567, *p* = 0.184) and parietal (*r* = 0.622, *p* = 0.135) lobes, and the whole brain (*r* = 0.478, *p* = 0.279); Fig. 4b).

#### Associations of ^11^C-BU99008 and VBM

BPM analysis in Aβ-positive patients showed reduced ^11^C-BU99008 uptake was correlated with reduced GM volume in the frontal and temporal lobes (Fig. 4c). ROI correlations showed the same correlation (Fig. 4d), showing strong correlations in the frontal (*r* = 0.808, *p* = 0.028), temporal (*r* = 0.935, *p* = 0.002), parietal (*r* = 0.833, *p* = 0.020) and occipital (*r* = 0.762, *p* = 0.047) lobes, as well as the whole brain (*r* = 0.901, *p* = 0.006).

#### Associations of ^11^C-BU99008 and ^18^F-florbetaben

BPM analysis in Aβ-positive patients described an inverse correlation of increased ^18^F-florbetaben uptake with reduced ^11^C-BU99008 uptake in regions such as the temporal lobe and the cingulate (Fig. 4e), whilst increased ^18^F-florbetaben uptake was positively correlated with increased ^11^C-BU99008 uptake in primary motor and primary sensory areas (Fig. 4g). ROI analyses showed that reduced ^11^C-BU99008 uptake was correlated with increased ^18^F-florbetaben uptake, particularly in the frontal (*r* = −0.780, *p* = 0.039), temporal (*r* = −0.779, *p* = 0.039) and occipital (*r* = −0.911, *p* = 0.004) lobe, as well as the whole brain (*r* = −0.798, *p* = 0.032; Fig. 4f).

Please note, the BPM and ROI results were not corrected for multiple comparisons due to the exploratory nature of this pilot study.

## Discussion

**Fig. 5:**
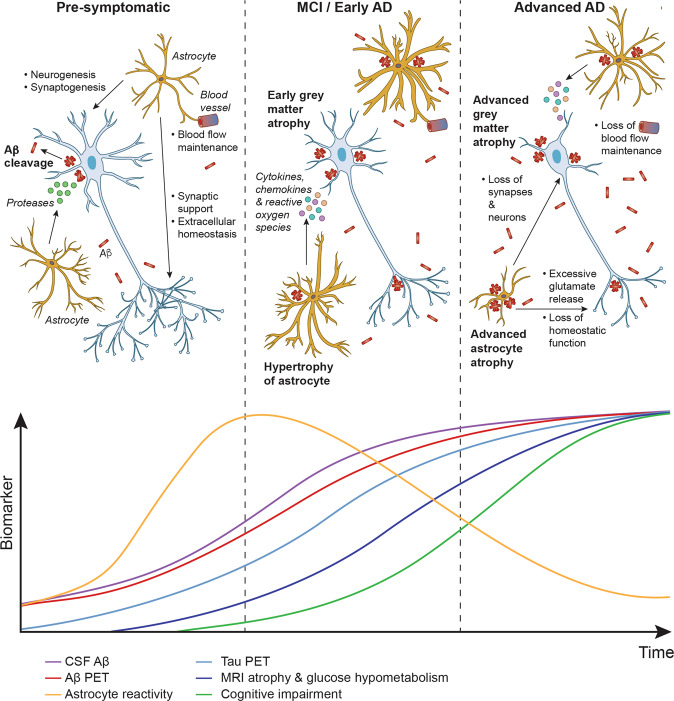
Schematic of the relationship between Aβ, astrocytes and neurons across the AD trajectory above a modified version of Jack et al. (2010) AD biomarker model. *Pre-symptomatic:* In healthy conditions, astrocytes have several roles in providing neuronal support that contributes to normal neuronal function. Accumulation of Aβ induces astrocyte reactivity, causing the astrocytes to become hypertrophic. Then, the neuroprotective reactive astrocytes release proteases that aid in the cleavage and removal of Aβ plaques. *MCI/Early AD:* Despite the efforts of the reactive astrocytes, Aβ continues to accumulate and eventually the high levels cause the reactive astrocytes to become neurotoxic, as they release cytokines, chemokines and reactive oxygen species. The switch from neuroprotective to neurotoxic contributes to the early stages of astrocyte and GM atrophy. *Advanced AD*: Astrocyte reactivity in cortical regions with early amyloid deposition, and therefore early astrocyte reactivity, will experience advanced astrocyte atrophy. This results in the loss of normal function (such as blood flow maintenance) and further new neurotoxic functions (such as excessive glutamate release), both contributing to glucose hypometabolism and advanced GM atrophy which further contribute to cognitive impairment.

In this study, we used the novel imidazoline receptor PET tracer ^11^C-BU99008 to test for evidence of a dynamic relationship between astrocyte reactivity and amyloid-associated neurodegeneration based on tissue hypometabolism and atrophy measured using ^18^F-FDG PET and structural MRI, respectively. We found evidence for increased astrocyte reactivity in Aβ-positive patients, as increased ^11^C-BU99008 uptake was observed primarily in frontal, parietal and occipital regions. Furthermore, these increases were greater in MCI than AD patients. Voxel-wise correlational analyses showed that lower ^11^C-BU99008 uptake in Aβ-positive patients was associated with hypometabolism in the parietal, temporal and frontal lobes, even though there was no correlation at ROI level. In addition, lower ^11^C-BU99008 uptake in Aβ-positive patients was associated with GM atrophy in frontal and temporal lobes both on a regional and voxel-wise basis. Finally, analyses of regional differences in the relationships between PET markers of Aβ-deposition and astrocyte reactivity displayed a striking heterogeneity. Previously, in the same cohort of patients, we evaluated positive correlations between amyloid and ^11^C-BU99008, and we observed that greater Aβ-deposition was associated with increased ^11^C-BU99008 uptake in primary motor and primary sensory cortical areas in the parietal cortex [19]. However, as astrocytes can have beneficial and detrimental effects, we performed further evaluation to explore positive and negative associations between amyloid and astrocyte reactivity. We observed greater Aβ-deposition was also associated with *decreased* ^11^C-BU99008 uptake in different localised regions in the rest of the cortices; particularly temporal and cingulate regions. These observations are consistent with a hypothetical model (illustrated in Fig. 5) in which astrocyte reactivity is maximal in earlier stages of pathological progression (when it may contribute to the clearance of Aβ plaques [34]), but, with greater Aβ deposition, astrocytes may progressively become dystrophic or transition to assume a neurotoxic phenotype [4], both of which are likely to accelerate neurodegeneration leading to brain atrophy and hypometabolism. Whilst there is regional, temporal and morphological variation with astrocyte reactivity across the AD spectrum [2], our results support previous research and a model which illustrates the rise and fall trajectory of gross levels of astrocyte reactivity through disease progression. This model is also supported by the results from previous studies using other astrocyte markers [21, 22, 35].

^11^C-BU99008 is a novel PET tracer that binds to I_2_-BS, expression of which is associated with astrocyte reactivity [36, 37]. Brain I_2_-BS is upregulated with healthy aging [38], and is further increased in AD [39]. The sensitivity and specificity of ^11^C-BU99008 to bind to I_2_-BS expressing reactive astrocytes has been further evidenced in a recent autoradiography study of AD brains where tracer uptake was more significant compared to cognitively normal brains [24]. In line with this, we have previously reported increased ^11^C-BU99008 uptake in the same cohort of cognitively impaired patients compared to healthy controls [19]. This corroborates with earlier studies using another PET marker of astrocyte reactivity, ^11^C-DED [35]. Interestingly, another ^11^C-DED study found increased binding in the frontal lobe in Aβ-positive MCI, but not AD, subjects compared to healthy controls [21], in line with findings in this present study as we observed increased ^11^C-BU99008 uptake in MCI subjects compared to AD subjects, particularly in the frontal, temporal and cingulate cortices. Both these findings agree with the hypothesis that astrocyte reactivity is predominantly an early event in the progression of AD pathology.

Astrocytes play an essential role in synapse formation along with the maturation of the synapses and synaptic pruning [40]. Astrocytes undergo morphological, molecular and functional remodelling in the presence of injury and disease states to become reactive astrocytes [2]. In the early stages, reactive astrocytes may have a neuroprotective role, aiding in the clearance of Aβ [3]. The spectrum of Aβ species mediating pathogenic changes in astrocytes is broad and complex, but it is hypothesised that Aβ oligomers can be involved in the primary pathogenesis of astrocyte reactivity [41, 42]. In support of this hypothesis, we found increased ^11^C-BU99008 uptake was associated with high levels of ^18^F-florbetaben uptake in primary motor areas, regions where amyloid deposition is to happen at a later stage of the disease and therefore would have recently developed. Increased ^11^C-DED binding has also been found in autosomal dominant AD patients early in their disease progression [43] with recent Aβ deposition [44]. In addition, a recent study demonstrated that plasma levels of glial fibrillary acid protein (GFAP), a marker of astrocyte reactivity, is an early marker of Aβ load, and was associated longitudinally with Aβ accumulation and cognitive decline [45].

Interaction of Aβ with reactive astrocytes has been proposed as a trigger for astrocytes to switch from a neuroprotective to a neurotoxic role. As astrocyte reactivity increases Aβ production [46], a positive feedback loop favouring the formation of neurotoxic, pro-inflammatory astrocytes is initiated. Reactive astrocytes exhibit beneficial and detrimental effects based on their reactivity profile. In mouse models of infection and stroke induced by lipopolysaccharide induction and middle artery occlusion, respectively, resting astrocytes polarise to become reactive astrocytes [47]. Neurotoxic astroglial phenotype is induced by cytokines secreted by activated microglia, which include complement factors (C1q), TNF-α and IL-1α [48]. This leads to astrocytes losing their ability to promote neuronal survival and outgrowth, synaptogenesis, and phagocytosis, and inducing the demise of neurons, oligodendrocytes and even astrocytes themselves. We found evidence of low astrocyte reactivity, perhaps due to astrocyte dystrophy in the presence of high Aβ load in this current study, as *decreased* ^11^C-BU99008 uptake was associated with high levels of ^18^F-florbetaben uptake in the temporal lobe, one of the earliest regions where Aβ deposition occurs [44]. This decreased ^11^C-BU99008 uptake in the temporal lobe was also associated with greater relative progression of amyloid-associated neuropathology, that is glucose hypometabolism and GM atrophy. However, it is important to note the evidence of an association between reduced ^11^C-BU99008 and ^18^F-FDG uptake is less strong as it was observed at a voxel-wise basis, but not on a regional basis. We propose this reduced ^11^C-BU99008 uptake in the temporal lobe region reflects astrocyte dystrophy [49], that eventually leads to astrocytic and neuronal atrophy [50, 51] and glucose hypometabolism [6] (Fig. 5). Our results are corroborated from previous findings of correlations between regional reductions in ^11^C-DED and ^18^F-FDG PET signals, which were associated with regionally more advanced ^11^C-PIB PET pathology in a longitudinal study of people with autosomal dominant AD or MCI [52].

There are obvious limitations to our study. First, only a small number of subjects were able to be imaged, requiring statistical analysis to be applied in a more liberal exploratory nature. However, while this is a pilot study, the explanatory power was enhanced by the design in which uptake of the three PET tracers and brain volume all were assessed in the same people. A second limitation was the cross-sectional design, which we acknowledge; however, *post mortem* pathology has the same limitation. Our results thus are better interpreted descriptively and as suggestive of a hypothetical model, rather than a strong, independent test. Nonetheless, the consistency of directions of effect observed in this study and the earlier ^11^C-DED PET studies [21, 52] provides compelling support for the hypothetical model presented (Fig. 5). That is, astrocyte reactivity occurs in response to early Aβ-deposition, aiding in the clearance of Aβ, but following interactions with high levels of Aβ the astrocytes become neurotoxic, contributing to reduced tissue activity and cell death that is associated with cognitive impairment. It also strengthens confidence in the earlier work, which otherwise suffers from uncertainties regarding the specificity of binding of ^11^C-DED in the brain [21]. Nonetheless, ^11^C-BU99008 can detect astrocyte reactivity with a greater sensitivity than ^11^C-DED [24], especially amongst higher levels of amyloid load [19, 53], and thus should be prioritised to take it forward.

In conclusion, this study supports neuropathological observations arguing that astrocyte reactivity with amyloid-related neuropathology is dynamic [54]. We have demonstrated in vivo with the novel PET tracer ^11^C-BU99008 that astrocyte reactivity is increased in regions presumed to represent earlier stages of pathological progression with low Aβ-deposition loads, and conversely relatively reduced in regions that show signs of more advanced disease progression with greater Aβ-deposition and atrophy. In the absence of molecular imaging markers intrinsically discriminating different reactive astrocyte phenotypes, our multi-modal imaging approach may allow relevant inferences to be made from the relative ^11^C-BU99008, ^18^F-FDG and ^18^F-florbetaben PET signals and brain volume sensitive MRI measures. Future, larger, longitudinal studies are needed to further test this dynamic model and, if supported, interventions developed to arrest progression of the neurotoxic phenotypic transformation of astrocytes in AD.

## Supplementary information

Supplementary Figures and Tables Legends

Supplementary Figure 1

Supplementary Table 1
