# Supplementary material for: Relationship between astrocyte reactivity, using novel 11C-BU99008 PET, and glucose metabolism, grey matter volume and amyloid load in cognitively impaired individuals
Source: Mol Psychiatry. 2022 Feb 7;27(4):2019–29. doi: 10.1038/s41380-021-01429-y (PMC9126819; doi:10.1038/s41380-021-01429-y)
Supplement: Supplementary file 1 — Supplementary Figures and Tables Legends [file 41380_2021_1429_MOESM1_ESM.docx]

**Supplementary Figure 1: Statistical Parametric Mapping (SPM) group analysis in MCI patients compared to AD patients.**

Rendered SPM results from two-sample Student’s t-tests (2-tailed) for differences in tracer uptake between MCI patients (N=5) and AD patients (N=6) with cluster threshold of p<0.05 and an extent threshold of 50 voxels. Coordinates for significant clusters following FWE-correction can be found in Supplementary Table 1. Colourbar units are contrast estimates representative of Z-scores.

**Supplementary Table 1: Results of group SPM analysis for ^11^C-BU99008, ^18^F-FDG, ^18^F-florbetaben PET and VBM.**

Clusters and coordinates displayed in MNI space, cluster threshold of p<0.05 (FWE-corrected) with extent threshold of 50 voxels.
