# Supplementary figures and images for: Relationship between astrocyte reactivity, using novel 11C-BU99008 PET, and glucose metabolism, grey matter volume and amyloid load in cognitively impaired individuals
Source: Mol Psychiatry. 2022 Feb 7;27(4):2019–29. doi: 10.1038/s41380-021-01429-y (PMC9126819; doi:10.1038/s41380-021-01429-y)

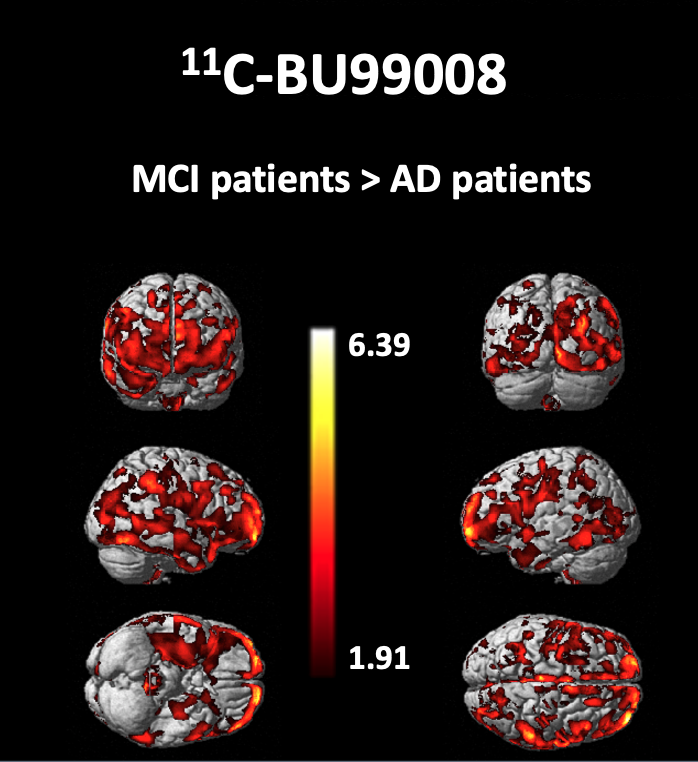

Supplement: Supplementary file 2 — Supplementary Figure 1 [file 41380_2021_1429_MOESM2_ESM.png]
