# Supplementary material for: Relationship between astrocyte reactivity, using novel 11C-BU99008 PET, and glucose metabolism, grey matter volume and amyloid load in cognitively impaired individuals
Source: Mol Psychiatry. 2022 Feb 7;27(4):2019–29. doi: 10.1038/s41380-021-01429-y (PMC9126819; doi:10.1038/s41380-021-01429-y)
Supplement: Supplementary file 3 — Supplementary Table 1 [file 41380_2021_1429_MOESM3_ESM.docx]

| Modality | Contrast | Coordinates | | | T-score | p_FWE-CORR_ | Region | Cluster size |
| --- | --- | --- | --- | --- | --- | --- | --- | --- |
|  |  | x | y | z |  |  |  |  |
| ^11^C-BU99008 | All Patients > Controls  MCI > AD patients | 57  37  41  9  28  23  44  20  35  34  47  8  60  -21  -11  -42  -17  -6  -48  -43  -51  -41  -52  13  -4  19  -7  41  -4  -13  18  6  15  22  -7  -4  -1  10 | 12  26  33  -43  64  15  23  35  10  43  43  -36  -4  -36  66  3  21  -33  -43  -32  24  -33  -18  -52  56  65  -28  60  39  68  -60  17  -69  -63  27  -1  -2  -64 | 37  41  18  79  14  52  35  49  60  26  3  79  38  77  4  52  65  73  44  65  38  61  23  2  17  -14  55  3  17  -7  18  43  31  26  36  48  40  5 | 5.64  4.95  4.67  4.35  4.24  3.95  3.94  3.90  3.88  3.82  3.82  3.82  3.61  4.55  4.42  4.14  4.13  3.75  3.64  3.51  3.40  3.35  3.32  6.39  5.92  5.82  5.72  5.51  5.47  5.43  5.27  5.17  5.00  4.87  4.77  4.74  4.69  4.54 | 0.001  0.006  <0.001 | Premotor cortex, R  Dorsolateral prefrontal cortex, R  Dorsolateral prefrontal cortex, R  Posterior parietal cortex, R  Anterior prefrontal cortex, R  Premotor cortex, R  Dorsolateral prefrontal cortex, R  Frontal eye fields, R  Frontal eye fields, R  Dorsolateral prefrontal cortex, R  Dorsolateral prefrontal cortex, R  Primary somatosensory cortex, R  Primary motor cortex, R  Primary somatosensory cortex, L  Anterior prefrontal cortex, L  Premotor cortex, L  Premotor cortex, L  Primary somatosensory cortex, L  Supramarginal gyrus, L  Primary somatosensory cortex, L  Frontal eye fields, L  Primary somatosensory cortex, L  Primary somatosensory cortex, L  Medial occipital cortex, R  Anterior prefrontal cortex, L  Anterior prefrontal cortex, R  Posterior parietal cortex, L  Anterior prefrontal cortex, R  Dorsal anterior cingulate cortex, L  Anterior prefrontal cortex, L  Medial occipital cortex, R  Frontal eye fields, R  Medial occipital cortex, R  Dorsal posterior cingulate cortex, R  Frontal eye fields, L  Premotor cortex, L  Ventral anterior cingulate cortex, L  Medial occipital cortex, R | 115,970  91,794    262,743 |
| ^18^F-FDG | All Patients < Controls | 20  43  52  23  60  38  21  -47  5  -20  44  -4  28 | -70  -56  -47  -68  -31  -67  -67  -67  -57  -71  -55  -33  -88 | 36  22  22  34  -2  18  40  11  9  32  -15  56  21 | 9.40  9.36  9.23  8.97  8.72  8.38  8.34  7.95  7.87  7.54  7.45  7.07  7.02 | <0.001 | Posterior parietal cortex, R  Angular gyrus, R  Angular gyrus, R  Posterior parietal cortex, R  Medial temporal gyrus, R  Lateral occipital gyrus, R  Posterior parietal cortex, R  Lateral occipital gyrus, L  Fusiform gyrus, R  Posterior parietal cortex, L  Fusiform gyrus, R  Posterior parietal cortex, L  Lateral occipital gyrus, R | 214,877 |
| VBM | All Patients < Controls | 48  51  28  21  44  24  35  40  35  48  57  26  61 | -38  -30  4  -8  -53  -12  -25  -50  15  -55  -3  -29  0 | -17  -19  -33  -15  -9  -33  -15  -15  -34  -2  -28  -26  -28 | 5.00  4.53  4.45  4.21  4.12  4.09  3.83  3.71  3.58  3.52  3.34  3.33  3.30 | <0.001 | Superior temporal gyrus, R  Inferior temporal gyrus, R  Parahippocampal gyrus, R  Hippocampus, R  Fusiform gyrus, R  Parahippocampal gyrus, R  Hippocampus, R  Fusiform gyrus, R  Temporal pole, R  Fusiform gyrus, R  Medial temporal gyrus, R  Fusiform gyrus, R  Medial temporal gyrus, R | 40,164 |
| ^18^F-florbetaben | Aβ+ Patients > Controls | -30  -23  -26  -59  23  -60  -28  18  3  -52  -2  -55  -8  -47  50  -6  39  -36  4  -7  -30  31  37  26  59  -3  -36  -47  -51  64  66  60  66  57  54  49  47  54  45  44  65  55 | -79  -79  -64  -58  -63  -59  -78  -78  -72  -76  -74  -62  -85  -76  9  36  53  56  51  36  45  63  61  47  -1  48  35  50  37  -29  -21  -4  -10  -60  -54  -44  -81  -42  -81  -76  -51  5 | 34  42  61  22  55  -14  30  44  43  1  43  -21  37  15  38  23  19  16  25  27  29  3  -2  29  37  22  39  0  8  36  23  38  -22  -9  -10  -27  -12  -27  -16  -18  10  -30 | 7.50  7.20  6.80  6.65  6.57  6.40  6.24  6.19  6.12  6.01  5.99  5.86  5.80  5.76  7.83  6.53  6.33  6.00  5.81  5.78  5.77  5.71  5.70  5.63  5.50  5.49  5.41  5.27  5.20  5.85  5.27  5.25  5.24  5.22  5.20  5.12  5.05  5.04  5.03  4.78  4.72  4.65 | <0.001  <0.001  <0.001 | Angular gyrus, L  Posterior parietal cortex, L  Posterior parietal cortex, L  Angular gyrus, L  Posterior parietal cortex, L  Fusiform gyrus, L  Angular gyrus, L  Posterior parietal cortex, R  Posterior parietal cortex, R  Lateral occipital gyrus, L  Posterior parietal cortex, L  Fusiform gyrus, L  Posterior parietal cortex, L  Lateral occipital gyrus, L  Premotor cortex, R  Dorsal anterior cingulate cortex, L  Anterior prefrontal cortex, R  Anterior prefrontal cortex, L  Dorsolateral prefrontal cortex, R  Dorsal anterior cingulate cortex, L  Anterior prefrontal cortex, L  Anterior prefrontal cortex, R  Anterior prefrontal cortex, R  Dorsolateral prefrontal cortex, R  Premotor cortex, R  Dorsolateral prefrontal cortex, L  Dorsolateral prefrontal cortex, L  Anterior prefrontal cortex, L  Dorsolateral prefrontal cortex, L  Supramarginal gyrus, R  Supramarginal gyrus, R  Primary motor cortex, R  Medial temporal gyrus, R  Fusiform gyrus, R  Fusiform gyrus, R  Inferior temporal gyrus, R  Lateral occipital cortex, R  Inferior temporal gyrus, R  Lateral occipital gyrus, R  Lateral occipital gyrus, R  Fusiform gyrus, R  Temporal pole, R | 115,994  105,945    48,055 |
